# Gender differences in eating disorders

**DOI:** 10.3389/fnut.2025.1583672

**Published:** 2025-06-02

**Authors:** Elena Ilaria Capuano, Angela Ruocco, Beatrice Scazzocchio, Giulia Zanchi, Camilla Lombardo, Annalisa Silenzi, Elena Ortona, Rosaria Varì

**Affiliations:** ^1^National Centre for Control and Evaluation of Medicines, Istituto Superiore di Sanità, Rome, Italy; ^2^Reference Center for Gender-Specific Medicine, Istituto Superiore di Sanità, Rome, Italy; ^3^Public Health Section, Department of Life, Health and Environmental Sciences, University of L’Aquila, L’Aquila, Italy

**Keywords:** eating disorders, men, gender differences, anorexia nervosa, bulimia nervosa, binge eating disorder

## Abstract

Eating disorders (EDs) are characterized by disturbances in eating behavior and occur worldwide, with a lifetime prevalence of 2 to 5%. Their etiology is complex and multifactorial, involving a complex interplay between genetic, biological, psychological, sociocultural, and environmental factors. They are more common among females than males and may be associated with medical and psychiatric complications, impaired functioning, and decreased quality of life. This narrative review aims at providing an updated contribution to the current understanding of gender differences in eating disorders (EDs) focusing on male population to foster more targeted and effective clinical interventions. A comprehensive review of the scientific literature was conducted by analyzing several major databases, including PubMed, PsycINFO, and Google Scholar. Only in recent years, there has been increased attention on the male population, revealing multiple differences between genders in terms of prevalence, onset, phenomenology, diagnosis, comorbidities, and outcomes of EDs. Moreover, the relationship between different sexual orientations and/or gender identities and EDs is an emerging field of study. Data suggest an increase in eating disorders (EDs) also among the male population underlines the importance that healthcare personnel of all specialties acquire basic competencies for adequately tackling these disorders in a gender perspective. In particular, prevention and early intervention, especially during critical developmental periods like puberty and adolescence, are crucial to avoid permanent damage. Future research and public health initiatives involving schools and families and targeting males should be addressed to promote a healthy relationship with food and body image, reduce stigma, and encourage people to seek help when needed.

## 1 Introduction

Eating disorders (EDs) are psychiatric pathological conditions commonly observed during early to late adolescence, a critical period for neural, physical, and psychological development. While the pathogenesis of EDs remains elusive, an apprehensive approach to weight, body shape, and eating behaviors plays a pivotal role in their onset. If untreated, EDs can lead to significant acute and long-term consequences (1) EDs are characterized by persistent disordered eating or eating-related behaviors that lead to impaired food consumption or intake and significantly impair physical health and psychosocial functioning (2). Both the Diagnostic and Statistical Manual (DSM-5) (2) and the International Classification of Diseases (ICD-11) (3) encompass six main eating disorders. These include the familiar diagnostic categories of anorexia nervosa (AN), bulimia nervosa (BN), and binge eating disorder (BED). In addition, three disorders regarded mainly as childhood disorders have been included, namely (i) the avoidant-restrictive food intake disorder (ARFID), characterized by a persistent failure to meet appropriate nutritional and/or energy needs, leading to significant weight loss, nutritional deficiency, dependence on enteral feeding or oral nutritional supplements, and marked interference with psychosocial functioning (4); (ii) pica, an eating disorder characterized by the persistent consumption of non-nutritive substances, such as dirt, clay, chalk, or paper, over a period of at least one month, at an age where this behavior is developmentally inappropriate (5); (iii) and rumination disorder, a condition characterized by the repeated regurgitation of food after eating, which is then either rechewed, re-swallowed, or spit out (6). The DSM-5 also provides sub-type qualifiers, severity indicators, and definitions of remission (7). Recent studies have shown that people with EDs exhibit unique somatic deficits, including increased or decreased sensitivity to internal body signals that inform them whether they are aroused, anxious, scared, or generally agitated. These individuals may use dysregulated eating strategies to reduce the physical sensations associated with emotional distress. Advances in understanding the causes of EDs could be beneficial for clinicians in treating their patients, helping them to stop feeling guilty about their bodies or behaviors (8). The importance of integrating sex and gender analysis in research studies is well established; the most important funding agencies, such as the European Commission and the National Institutes of Health, have signed policy changes concerning this (9). The sex of an individual is defined by a set of biological characteristics present at birth, such as sex chromosomes, gonads, genitalia, and sex hormones. A person may be born with typically male, female, or, more rarely, sex characteristics that are not attributable to those typically considered male or female (intersex individuals). The term ‘gender,’ which should not be confused with ‘sex,’ refers to the socially and culturally defined characteristics that distinguish masculinity from femininity, encompassing the complex of norms, roles, and relationships between individuals defined as men and women. Gender is therefore a sociocultural construct and, as such, varies across societies and can change over time (10). It is also well recognized that the manifestation of eating disorders might vary between men and women (3). Although recent investigations have begun to explore EDs in the male population, most of the studies, to date, have been conducted on female samples, reinforcing the misconception that these disorders affect only women. Consequently, EDs in males are often underdiagnosed, poorly treated and understood, leading to symptoms overlooked and recognized only when they have become particularly severe and disabling (11). The majority of the reports on EDs show poor gender-specific results, especially because of small samples or the exclusion of males (12). Only in recent years, there has been increased attention on the male population, revealing multiple differences between genders in terms of prevalence, onset, phenomenology, diagnosis, comorbidities, and outcomes of EDs. Studies on risk factors have also shown significant differences between males and females, particularly concerning the psychological variables involved in the etiology of EDs. Historically, eating disorders have been perceived as predominantly affecting females, particularly adolescents and young adults, cisgender, heterosexual, of white ethnicity, and from affluent social classes (13, 14). This generalization has deep historical roots, due to the association between AN and hysterical disorder, that were considered pertinent to the women (15), as also described for the first time by Rudolph *M. bell* in 1985 in the “Anorexic Saints.” These perceptions have been reinforced over the decades by mass culture, which has long identified the models promoted by the fashion and entertainment industries as sources of EDs, particularly among adolescent girls. Only recently, it has emerged that eating disorders can affect the entire population, with different peculiarities and variations depending on several factors such as gender, sexual orientation, and age (16). The late detection of EDs in both men and women represents a significant health risk. In contrast, an early diagnosis and a consequent appropriate intervention are crucial for improving outcomes in these potentially life-threatening disorders. The WHO’s Comprehensive Mental Health Action Plan 2013–2030 recognizes the crucial role of mental health in achieving health for all people, establishing some priorities (17). For instance, among others, this plan tries to strengthen information systems, evidence, and research for mental health. In this sense, our review aims to provide an updated contribution to the current understanding of gender differences in EDs, with a specific focus on the male population, to facilitate the development of more targeted and effective clinical interventions. Boys are often presumed to underreport the problem due to societal perceptions that these disorders predominantly affect girls, as well as the widespread misconception that disordered eating behaviors are exclusive to females. Furthermore, existing diagnostic criteria for eating disorders frequently fail to identify disordered eating patterns more commonly observed in males. Considering that mid to late adolescence is a peak period of eating disorders and their symptoms, knowing and understanding the proportion of disordered eating among young males is a crucial issue. Consequently, the purpose of this review is addressed to fill this gap.

## 2 Methodology

A comprehensive narrative review of the scientific literature was conducted by analyzing several major databases, including PubMed, PsycINFO, and Google Scholar up to year 2025. The keywords used in the search included: “gender,” “males,” “eating disorder,” “anorexia nervosa (AN),” “bulimia nervosa (BN),” “binge eating (BED),” and “muscle dysmorphia.” In an initial search, 452 papers were identified considering all the keywords selected. A total of 111 articles were evaluated for detailed analysis, with a preference given to systematic reviews, meta-analyses, and controlled trials that explicitly examined eating disorders in men, addressing aspects such as prevalence, onset, phenomenology, diagnosis, comorbidities, and outcomes. Of these, 45 studies are discussed in the present review.

## 3 Epidemiology of eating disorders

The etiology of eating disorders is very complex and arises from the intersection of many risk factors. Although the prevalence varies according to study populations and definitions used, it is recognized that eating disorders are common in adolescents and even more common in young adults. Based on the DSM-5, the prevalence of eating disorders in children and adolescents (aged 11–19 years) has been stated to be between 1.2% (boys) and 5.7% (girls), with increasing incidence over recent decades (18). Eating disorders can affect individuals of all ages, genders, sexual orientations, ethnicities, and geographies. A systematic review of studies based on prevalence in the general population, published between 2000 and 2018, using validated diagnostic instruments, estimated the worldwide lifetime prevalence of EDs in men to be 0.2% for AN, 0.6% for BN, 1.0% for BED, and 3.6% for disorders not otherwise specified, and not meeting the criteria for the complete picture of other categories (19, 20). Epidemiological data for AN and BN indicate that in adolescents (10–19 age range) and young adults (20–40 age range) in Western countries, those EDs are among the most common health issues, showing one of the highest mortality rates among psychiatric illnesses (21). In Western settings, a substantial proportion of young people reported to be affected by an eating disorder. Overall, 5.5–17.9% of young women and 0.6–2.4% of young men have experienced an ED by early adulthood. Considering the entire lifetime, AN was reported in 0.8–6.3% of women and 0.1–0.3% of men; BN in 0.8–2.6% of women and 0.1–0.2% of men; BED in 0.6–6.1% of women and 0.3–0.7% of men, other specified feeding or eating disorders in 0.6–11.5% of women and 0.2–0.3% of men, and unspecified feeding or eating disorders in 0.2–4.7% of women and 0–1.6% of men (21, 22). Emerging studies from Eastern Europe, Asia, and Latin America show similar high prevalence. During the COVID-19 pandemic, the incidence of EDs has further increased (22). It is noteworthy that recent data from the COPSY Study (23) have shown an increase in EDs symptoms in German boys as a result of the COVID-19 pandemic. On the contrary, in the UK, EDs declined in boys and rose in girls as a result of the pandemic (24). Italian epidemiological data confirm that both AN and BN are significant public health concerns (25). In Italy, the age range for the onset of both AN and BN is 15–19 years, with a trend toward increasingly earlier onset in recent years. Although the overall incidence rate of AN is considerably stable over the past decades, the incidence among younger people (aged < 15 years) has increased. However, it is unclear whether this reflects earlier detection or earlier age of onset. Nevertheless, there are implications for future research into risk factors and for prevention programs. The lifetime prevalence rates of AN might have grown to 4% in females and 0.3% in males, as well. Concerning BN, there has been a decline in the overall incidence rate over time, but up to 3% of females and more than 1% of males suffer from this disorder during their lifetime. While epidemiological studies from Western countries, in the past, mainly focused on young females, AN and BN are reported worldwide among males (26). According to Italian Ministry of Health’s data, the estimated incidence of AN is at least of 8 to 9 new cases per 100,000 people per year among women, between 0.02 and 1.4 new cases per 100,000 people per year among men. Clinically, the symptoms and course tend to be similar across genders. Males with AN share many symptomatic similarities with females, although they often have a premorbid history of overweight and typically experience a later age of onset, starting from late adolescence onward (27, 28). Another significant data point is lifetime prevalence. However, in general, in Western settings the prevalence of EDs among men has increased dramatically over the last two decades and while it has been commonly cited that 10% of clinical ED cases occur in men (29), more recent data suggest that this number may be as high as 25% (30). Additionally, these figures are likely an under estimation of actual prevalence rates as low numbers of men with EDs present to health services, in part, due to lack of public awareness and stigma (31, 32). In Western settings adolescents and young adults are at higher risk, with AN starting earlier than BN or BED (33, 34). Vo et al. in an observational study, comparing DSM-IV and 5 criteria, found that the cases of specified eating disorders (AN, BN, BED) were increased in preadolescent patients, on the contrary those of disorders not otherwise specified were reduced, highlighting the impact of these revisions on rates of eating disorder diagnoses (35). Also during adolescence, according to DSM-5 diagnostic criteria, the prevalence of EDs increases in the male population, estimated approximately at 1.2% at age 14, 2.6% at age 17, and 2.9% at age 20. For BN, the prevalence rates are 0.4, 0.7, and 1.6%, respectively (36). Considering the gender prevalence of eating disorders during adolescence, based on the criteria of the latest edition of the DSM, 1.2% of males develop AN or BN compared to 5.7% of females. BED is the most common diagnosis among young males, with an estimated prevalence of around 2% (versus approximately 3.5% in females) (37). In the report “Appropriate Clinical, Structural, and Operational Practices in the Prevention, Diagnosis, and Treatment of Eating Disorders” (38), researchers, discussing epidemiological data, emphasize the need for further studies on prevalence and incidence of EDs by evaluating large population samples. They highlight that this issue was already discussed during the Italian National Consensus Conference on Eating Behavior Disorders in Adolescents and Young Adults in Rome (39). Despite the complexity of integrating all ED prevalence data, the most recent studies confirm that EDs are highly prevalent worldwide, especially in women, but growing in men. A summary of the findings from main studies is shown in Table 1.

**Table 1:** A summary of the findings from main studies.

Author, publication year	Objective	Study	Results
Allen et al. (36)	Prevalence of AN and BN in Australian adolescent’s male and female adolescents (14–20 years).	Observational Study	AN: 1.2% (age 14), 2.6% (age 17), 2.9% (age 20); BN: 0.4% (age 14), 0.7% (age 17), 1.6% (age 20).
Smink et al. (37)	Prevalence of AN, BN, and BED in adolescents in western setting.	Review	AN or BN: 1.2% (boys), 5.7% (girls); BED: 2% (boys), 3.5% (girls).
Javaras et al. (33)	Incidence of AN and OED from 1987 to 2009 (when individuals were 8–30 years old) for a cohort of 2.3 million individuals (48.7% female) born from 1979 to 2001 in Sweden.	Observational Study	AN peak age incidence in girls (14–15 years), in boys (12–13 years). OED peak age incidence in girls (16–17 years), and boys (14–15 years).
Donini et al. (25)	Italian epidemiological data on eating disorders.	Observational Study	AN: 8–9 new cases per 100,000 people (women), 0.02–1.4 new cases per 100,000 people (men).
Galmiche et al. (19)	Global prevalence of eating disorders in general population (under and over the age of 18) in Western countries.	Systematic review	Lifetime prevalence of EDs: 8.4% women, 2.2% men. AN: 1.4% women, 0.2% men BN: 1.9% women, 0.6% men; BED: 2.8% women, 1.0% men.
Van Eeden et al. (26)	Epidemiology of AN and BN in terms of incidence, prevalence and mortality on all age groups worldwide.	Review	AN and BN occur among girls and boys of all age groups worldwide and are associated with an increased mortality risk.
Silén et al. (22)	Prevalence of eating disorders in women and men in western setting.	Review	AN: 0.8–6.3% women, 0.1–0.3% men; BN: 0.8–2.6% women, 0.1–0.2% men; BED: 0.6–6.1% women, 0.3–0.7% men.
Boni et al. (30)	Prevalence of eating disorders in men in western setting.	Review	1.2% of cases at age 14; 2.6% of cases at age 17; 2.9% of cases at age 20. 0.16–0.3% of AN, 0.1–0.5% of BN and 1.1–3.1% of BED.
Napp et al. (23)	Differences in the prevalence of eating disorder symptoms among children and adolescents (11–17 years) in Germany before and after the onset of the COVID-19.	Longitudinal study	Increase in eating disorder symptoms in boys. 17.18% girls; 15.08% boys.
Trafford et al. (24)	Incidence on eating disorder among young people (10–24 years) in the UK in the 2 years following onset of the COVID-19 pandemic.	Observational study	Incidence of eating disorders decline in boys and increase in girls higher than expected: girls: 42.4% (13–16 years), and 32% (17–19 years); boys: 22.8%.
López-Gil et al. (18)	Prevalence of disordered eating in children and adolescents (11–19 years) from 16 countries.	Systematic review	1.2% (boys) and 5.7% (girls), with increasing incidence over recent decades.

AN, Anorexia nervosa; BN, Bulimia nervosa; BED, Binge eating disorder; OED, Other eating disorders.

## 4 Gender differences in eating disorders

Eating disorders do affect also men, but phenotypic differences between women and men, generally not considered in the diagnostic instruments, can hamper an early diagnosis. This may have effects on care provision and treatment results, that in addition have not been sufficiently studied because of the lack of data. Phenomenologically, males tend to hide the disorder behind strict dietary regimens and intense sports practices. They are often involved in physical activities emphasizing muscularity and are more inclined than females to engage in excessive physical exercise (40). Consequently, EDs in males are often associated with disorders such as “muscular dysmorphia” or “bigorexia” (41). Research attests to a higher prevalence of EDs among young athletes (42). Additionally, males use vomiting, diuretics, and laxatives less frequently than females (25% vs. 50%), preferring exercise or fasting as compensatory practices following binge eating. These factors can hinder the recognition of the severity of the ED by the caregivers or delay the patient’s request for help, often arriving to clinical observation in an advanced state of malnutrition (43, 44). Men with EDs often delay help-seeking and tend to present later in the course of their illness when ED behaviors and symptoms are more severe and less tractable to intervention (45, 46). When men with EDs do seek treatment, there is an additional risk that their symptoms may go undiagnosed by health professionals due to the false notion that EDs only affect women and girls (47, 48). The double stigmatization, the shame of having a disease considered typically female on one hand, and the conflict with masculine identity on the other one, make it unlikely that men refer their ED symptoms in the medical and therapeutic context. Consequently, the challenge for doctors to probe the presence of EDs in men, to mention them when suspected, and to drive men to suitable treatment is even more demanding with respect to women (49). Studies have identified some sex-specific genetic risk factors. For example, women relatives of men with AN have a higher risk of developing the same ED. Twin studies, including same-sex twins and opposite-sex twins, report that about 50% of the genetic risk for EDs is shared. Most interestingly, female members of male–female twin pairs had a consistently higher genetic risk than males for developing an ED. Hormonal maturation is significant since the risk for females in these studies was not detectable until after puberty (50). Overall, the results of these studies indicate the presence of a gap in the knowledge and the need to investigate the specific pathways that may lead to an ED with reference to different sexual orientations and/or gender identities, for which the data seem to suggest the existence of different etiopathological mechanisms.

### 4.1 Anorexia nervosa

According to the DSM-5 (DSM-5, 2015) the diagnostic criteria for AN are:

- Restriction of calorie intake relative to need, leading to significantly low body weight in the context of age, sex, developmental trajectory, and physical health. Significantly, low body weight is defined as less than the minimum average weight or, for children and adolescents, less than the minimum expected weight.
- Intense fear of gaining weight or becoming fat or persistent behavior that inter-feres with weight gain, even if significantly low.
- Disturbance in how one’s weight or body shape is experienced, undue influence of weight or body shape on self-esteem, or persistent lack of recognition of the severity of the current low body weight.

The first clinical case of male AN was described in 1689, when the English physician Richard Morton reported the story of a 16-year-old boy suffering from what he called “nervous consumption.” The patient had a “total lack of appetite” with no organic symptoms, leading the physician to hypothesize that the problem was nervous rather than organic (51). After these early cases, males with EDs were marginalized for many centuries, considered “rare,” and largely forgotten until 1972, when Peter Beaumont and colleagues studied AN in men. The diagnosis of EDs in men has long been hindered by the inadequacy of the leading classification criteria, which were designed to identify the disorder primarily in women. In the case of AN, the criterion of amenorrhea (absence of at least three consecutive menstrual cycles), an indicator of prolonged malnutrition in women, did not have valid counterpart for males regarding neuroendocrine alterations. Even the body mass index (BMI), used to assess weight adequacy, is not a reliable indicator for males, as a normal weight condition can conceal an ED in men. Therefore, the DSM-5 has made specific changes to the diagnosis of EDs to promote a more accurate framework for males. Notably, the elimination of the amenorrhea criterion for AN and related modifications to the classification of Eating Disorder Not Otherwise Specified (EDNOS) have allowed for greater diagnostic accuracy and reduced the frequency of residual diagnoses in the male population. The EDNOS classification, which included partial forms of AN and BN, often provided little descriptive information on the characteristics of EDs in the general population (37, 52). Recently, a case–control study compared the risk of death between men and women over a time period of 6–12 years (53). The age and sex standardized death risk was increased for both men and women with AN and EDNOS. The authors found death rates in men with BN and EDNOS, not significantly different to those in women. However, this finding can be explained with the fact that the study was insufficiently powered because of the too small case numbers in men enrolled in the study (188 men versus 5,296 women). Moreover, the survival period was shorter for men with AN compared to women.

### 4.2 Bulimia nervosa

According to the DSM-5 (DSM-5, 2015) the diagnostic criteria for BN are:

- Recurrent episodes of binge eating, as characterized by both:

Eating, within any 2-h period, an amount of food that is definitively larger than what most individuals would eat in a similar period of time under similar circumstances.

A feeling that one cannot stop eating or control what or how much one is eating.

- Recurrent inappropriate compensatory behaviors to prevent weight gain, such as self-induced vomiting, misuse of laxatives, diuretics or other medications, fasting, or excessive physical activity.
- Binge eating and inappropriate compensatory behaviors occur on average at least once a week for three months.
- Self-esteem is unduly influenced by body shape and weight.
- Impairment does not occur exclusively during episodes of AN.

Few studies have investigated the incidence of BN; in the US, a registry study provides indications of an increased prevalence of ED behaviors in men from marginalized ethnic and cultural groups, especially as regards bulimia (lifetime prevalence in men from a Latin-American background: 1.73% versus Western men: 0.08%) (54). A review of culture-comparison studies concluded that weight control behavior and binge eating largely affect men from marginalized ethnic groups (55). A further review showed that rates of EDs in homosexual or bisexual men are higher than in heterosexual men (56). According to a meta-analysis, symptoms of an ED are more strongly pronounced in trans men than in trans and cis women (78). However, the sociocultural factors that differently affect the risks for EDs in men and women are not sufficiently known. The incidence of BN in Italy is estimated to be at least 12 new cases per 100,000 people per year for females and about 0.8 new cases per 100,000 people per year for males (57). These data further support the rise in ED in the male population. Furthermore, some researchers believe these figures are still underestimated. This may be partly due to the absence of specific diagnostic criteria for males, making it difficult to diagnose, resulting in symptoms often being considered sub-threshold, poorly understood, or overlooked unless they become particularly severe (46). As further confirmation of this, subjects who fit the diagnosis of muscle dysmorphia are not correctly classified as having an ED because this condition has not yet been officially recognized as part of the ED spectrum but it is classified, instead, as a body dysmorphic disorder. Another reason for the underestimation could be that EDs have traditionally been considered a predominantly female condition, thus men are reluctant to admit they suffer from it. Consequently, there is often a significant delay between the onset of symptoms and the start of treatment. Therefore, accurate diagnostic evaluation is critical, as it allows for the proper estimation and classification of ED in males.

### 4.3 Binge eating disorder

BED is primarily characterized by large binge episodes, marked by a sense of loss of control over eating. The criteria identified by the DSM-5 manual can be summarized as follows:

- A feeling of loss of control during the binge episode.

Eating within a limited period (e.g., two hours) an amount of food that is significantly larger than most individuals can eat at the same time under similar circumstances.

Recurrent binge episodes associated with three (or more) of the following features.

- Eating much more rapidly than usual.
- Eating until feeling uncomfortably full.
- Eating large amounts of food when not physically hungry.
- Eating alone due to embarrassment over how much one is eating.
- Feeling disgusted with oneself, depressed, or very guilty afterward.
- Marked distress regarding binge eating.
- The frequency of binge eating, on average, is at least twice a week for six months.
- Absence of inappropriate compensatory behaviors as seen in bulimia nervosa.

BED, unlike AN and BN, affects equally males and females who aged between 30 and 40. The worldwide prevalence of BED for the years 2018–2020 is estimated to be 0.6–1.8% in adult women and 0.3–0.7% in adult men (58). According to experts, 3.5% of women and 2% of men have experienced this disorder during their lifetime (57). BED is commonly associated with obesity and with somatic and mental health comorbidities. People with BED experience considerable burden and impairments in quality of life, at the same time (58). BED is an important public health problem because of its close relation to other medical and psychiatric disorders, especially obesity and depression, thus developing more effective BED treatments is an urgent need (59). According to several studies on BED (60, 61) males exhibit a tendency toward a higher lifetime maximum weight and elevated Body Mass Index (BMI) compared to females. Conversely, females demonstrate significantly higher scores on measures of eating disorder-specific psychopathology, including greater concern regarding body shape and weight, and elevated levels of depression. The aforementioned research, however, does not identify significant gender-based differences in the frequency or severity of binge eating episodes, nor in the prevalence of other psychiatric comorbidities (with the exception of the higher depression scores observed in females) (62). BED frequently presents as a less conspicuous condition compared to other eating disorders such as anorexia nervosa and bulimia nervosa, which garner greater attention. This relative invisibility can contribute to an underestimation of its severity and the consequent need for treatment.

## 5 The gender-expansive population and ED

Some studies have included LGBT+ individuals, who often report a higher prevalence of psychiatric diseases and EDs. LGBT+ is the acronym, derived from the English Lesbian, Gay, Bisexual, and Transgender which, since the 1990s, has been used to define people who do not identify themselves as cisgender and/or heterosexual but rather as Lesbian, Gay, Bisexual, and Transgender. In order to make the acronym more inclusive, a + symbol has been added to refer to intersex, gender diverse, genderqueer, genderfluid, and asexual people. Their experience of systematic familial and social discrimination has been associated with multiple dysregulated eating phenotypes such as BED, fasting, and vomiting. Females in LGBT+ community often attempt to suppress menstruation through extreme caloric restriction or excessive exercise. However, no systematic research has been conducted on the genetic risk of EDs among LGBT+ individuals. Scientists presume that the genetic influences on EDs acting on these individuals are the same as in cisgender people, but the sociocultural risk factors have a significant impact. A recent study by Nagata et al. (56) was one of the first to examine EDs in the gender-expansive population. The researchers define “gender-expansive” as the spectrum of gender identities that do not fit within the binary system (man or woman). This can include individuals who identify as agender (not identifying with any gender), genderqueer or non-binary (referring to those whose gender identity does not conform to the binary conception of gender), pangender (identifying with multiple or all genders), and gender fluid (a gender identity that fluctuates among genders depending on the time or other circumstances). Gender-expansive is distinct from transgender, which refers to individuals whose gender identity and/or gender expression differ from the typical associations with the sex assigned at birth (63). A recent study suggests that gender-expansive individuals experience higher levels of psychological distress, less social support, encounter more bullying, and have worse psychological well-being outcomes than transgender and cisgender people (64). This study aimed to establish community norms for one of the most commonly used instruments in ED research, the Eating Disorder Examination Questionnaire (EDE-Q) (65). Researchers evaluated 998 gender-expansive participants from the Population Research in Identity and Disparities for Equality (PRIDE) study, a longitudinal study of adults living in the United States who identify as a sexual and/or gender minority. The average age of participants was 29 years; 79% was identified as white, 63% had a college degree or higher. The results are as follows: 23% of participants reported dietary restriction, 12.9% reported binge eating, 7.4% reported excessive exercise, 1.4% reported self-induced vomiting, 1.2% reported laxative abuse, and 13.8% had been diagnosed with an ED by a health care provider. There were no significant differences in eating attitudes or dysregulated eating behaviors between gender-expansive individuals and cisgender. Gender-expansive individuals reported lower scores for dietary restriction and shape concern than transgender women; higher scores of concern about eating, weight, and fitness compared to presumed cisgender men; and lower scores of concern about fitness than presumed cisgender women. Before this study, there were no established community standards for EDE-Q for gender-expansive people, who are rarely included in ED research. This lack of inclusion has made difficult, if not impossible, to access evidence-based treatments. When treatments are studied only on a specific population subgroup, it is impossible to know if the same treatments are equally effective for people outside the studied group or if modifications to the treatments are necessary. This bias in research leads to biases in health care where marginalized groups are unable to receive scientifically proven effective treatments. Gender minority groups face unique stressors that increase the risk of EDs, particularly concerning the link between body image and gender identity. Researchers hypothesize that gender-expansive individuals may be less influenced by binary gender body ideals compared to transgender or cisgender individuals. Gender-expansive individuals constitute a distinct group with unique implications for ED symptoms and treatment. Further research is needed to better understand the complex nature of such symptomatology in this population and in other marginalized groups (56) (Supplementary Appendix).

## 6 Discussion and conclusion

Despite the increased incidence of EDs in the male population over the past 15–20 years, only recently researchers have focused on gender differences, resulting in a partial rebalancing of the classical female-centric perspective. The changes introduced by DSM-5 seem to have favored a more accurate diagnosis of EDs in men, whose symptoms tend not to fit into the stricter categories of previous classifications. While these changes have led to an improvement in diagnostic criteria positively impacting the identification of the disorder in men, many areas related to gender differences in phenomenology, onset, symptom manifestations, comorbidity, and outcome remain still underexplored. Over the past thirty years, the scientific debate around EDs has seen significant developments regarding sex and gender-related issues, such as the removal of the amenorrhea criterion from the DSM-5 for the diagnosis of AN (66) and the growing body of literature and clinical and media attention on the subject. However, the issue remains complex and challenging. The influence of sex and gender on human health and disease is an increasingly recognized area of research, yet it remains underappreciated and insufficiently addressed in clinical practice. Recognizing and understanding the distinct nutritional requirements and dietary behaviors associated with gender is essential for clinicians aiming to provide tailored nutritional interventions, particularly in pediatric populations (67). Dietary recommendations should be gender-specific from early childhood and integrated into the health management of children and adolescents, as nutritional needs and eating patterns can diverge significantly between boys and girls due to both biological and socio-cultural factors (68). Dietary habits and adherence to dietary patterns are strongly influenced by environment, social norms, and ethnicity, particularly in adolescence. Recent studies have shown gender roles in the establishment of dietary patterns and eating habits. Addressing gender-specific factors and societal influences is crucial in designing effective nutrition education programs and interventions targeting adolescents (69). Promoting healthier dietary patterns and body image perceptions requires a comprehensive approach that considers individual, familial, societal, and environmental factors (70). Moreover, the relationship between different sexual orientations and/or gender identities and eating disorders is an emerging and complex field of study that needs to be studied in depth (71). In conclusion, given data suggesting that medical psychologists and nutritionists will increasingly encounter male patients with EDs in their clinical practice soon, it is essential to expand the understanding of the disorders in men, considering a gender perspective. Particular attention should be paid to crucial developmental periods such as puberty and adolescence, where early identification and prevention of these disorders are of primary importance within a conceptual framework that considers the reciprocal influence between psychological vulnerability and sociocultural factors (72). Early onset is indeed a critical issue, as malnutrition can cause permanent damage to organs and tissues that have not yet completed their development (73). For this reason, in recent years, clinicians have emphasized the utility of early interventions and continuity of care during this crucial phase of personal development (25, 57). Early interventions are also highlighted in the NICE Guidelines 2017, emphasizing the need to consider pathology and adopt a multidisciplinary approach that addresses comorbidities and any associated pathological dependencies. It is well documented that eating disorders are underrecognized and undertreated in males (56, 66, 74, 75). Men with eating disorders often experience delays in identification, diagnosis, and referral to care, and frequently present with more severe illness. This issue is partly attributable to a lack of sex and gender specific medical treatment guidelines for healthcare professionals (76). Despite growing awareness of this gap, current treatment recommendations from leading organizations such as the American Society for Adolescent Health and Medicine and the National Institute for Health and Care Excellence do not yet incorporate sex and gender-specific considerations (77). The urgency to address this shortfall is underscored by the fact that males with eating disorders face significant medical complications, elevated mortality rates, and are being hospitalized at increasing rates. Persistent stereotypes that eating disorders primarily affect females contribute to underdiagnosis and undertreatment in men, as both patients and clinicians may overlook symptoms in males. Furthermore, men may be less likely to seek help due to stigma and societal expectations, which can delay intervention and worsen outcomes. In conclusion, our review highlights that studies focusing on males remain limited, frequently lack comparability, and often fail to encompass the full spectrum of eating disorders. To address these gaps, it is crucial to develop sex and gender-specific screening tools, enhance clinician awareness, and integrate male-centered perspectives into research and clinical practice particularly regarding the prevalence, onset, phenomenology, diagnosis, comorbidities, and outcomes of eating disorders. The implications for practice are significant due to the need to improve secondary prevention tools and healthcare practice management addressed to male population. Disseminating these findings and recommendations for implementation will improve a potentially growing disparity in the treatment of eating disorders among non-identifying females in our global society.
