# Supplementary material for: Gender differences in eating disorders
Source: Front Nutr. 2025 Jun 2;12:1583672. doi: 10.3389/fnut.2025.1583672 (PMC12171451; doi:10.3389/fnut.2025.1583672)
Supplement: Supplementary file 1 [file Data_Sheet_1.docx]

**Appendix**

AGENDER: literally "genderless", this term refers to people who do not identify with any gender.

ASEXUAL: person who is not sexually attracted toward other people of any gender. Contrary to what might be thought, this does not mean that asexual people do not enjoy sexuality.

BINARY: adjective used to state the existence of two genders: male / female.

BISEXUAL: adjective used to refer to a person who is emotionally and / or sexually attracted to people of both genders.

CISGENDER: adjective referred to people whose gender identity matches the sex assigned at birth.

GAY: adjective indicating a man in love and/or sexually attracted to another man.

GENDER IDENTITY: an intimate and profound sense of belonging to one gender, male, female or an alternative one. Gender identity may or may not match the gender assigned at birth or the primary or secondary sexual characteristics.

GENDER EXPRESSION: refers to how a person enacts or expresses their gender in everyday life and within the context of their culture and society. Expression of gender through physical appearance may include dress, hairstyle, accessories, cosmetics, hormonal and surgical interventions as well as mannerisms, speech, behavioral patterns, and names.

GENDERQUEER: expression referring to people whose gender identity does not conform to the binary view of gender. Genderqueer people can define themselves as both man and woman); neither as a man nor as a woman; they can fluctuate between genders (genderfluid); or embody a third gender.

LESBIAN: adjective indicating a woman in love and/or sexually attracted to another woman.

LGBT+: an acronym of Anglo-Saxon origin used to indicate lesbian, gay, bisexual, transgender people. The + symbol is added to include other sexual minorities such as intersex, genderqueer, genderfluid, asexual people, etc.

PANGENDER: the pangender person (literally "all genders") lives in a multiplicity of simultaneous genders. Furthermore, the pangender person can experience a flux / alternation of genders.

TRANSGENDER: umbrella term used to describe people whose gender identities and/or gender expression are not what is typically expected for the sex to which they were assigned at birth.
